# Gastroprotective [6]-Gingerol Aspirinate as a Novel Chemopreventive Prodrug of Aspirin for Colon Cancer

**DOI:** 10.1038/srep40119

**Published:** 2017-01-09

**Authors:** Yingdong Zhu, Fang Wang, Yantao Zhao, Pei Wang, Shengmin Sang

**Affiliations:** 1 Laboratory for Functional Foods and Human Health, Center for Excellence in Post-Harvest Technologies, North Carolina Agricultural and Technical State University, North Carolina Research Campus, 500 Laureate Way, Kannapolis, NC 28081, USA; 2 Department of Gastroenterology, General Hospital of Ningxia Medical University, Yinchuan 750004, P.R. China

## Abstract

A growing body of research suggests daily low-dose aspirin (ASA) reduces heart diseases and colorectal cancers. However, the major limitation to the use of aspirin is its side effect to cause ulceration and bleeding in the gastrointestinal tract. Preclinical studies have shown that ginger constituents ameliorate ASA-induced gastric ulceration. We here report the design and synthesis of a novel prodrug of aspirin, [6]-gingerol aspirinate (GAS). Our data show that GAS exerts enhanced anti-cancer properties *in vitro* and superior gastroprotective effects in mice. GAS was also able to survive stomach acid and decomposed in intestinal linings or after absorption to simultaneously release ASA and [6]-gingerol. We further present that GAS inactivates both COX-1 and COX-2 equally. Our results demonstrate the enhanced anticancer properties along with gastroprotective effects of GAS, suggesting that GAS can be a therapeutic equivalent for ASA in inflammatory and proliferative diseases without the deleterious effects on stomach mucosa.

Ginger (*Zingiber officinale*) is widely used as a spice and an ingredient in traditional herbal medicine. The rhizome of ginger has been shown to ameliorate symptoms such as inflammation, rheumatic disorders, gastrointestinal discomforts, and nausea and vomiting associated with pregnancy^1^. Considerable evidences in preclinical and clinical studies have been reported regarding the gastroprotective effects of ginger root extracts or ginger essential oils^2^,^3^,^4^,^5^. Recently, ginger powder has been shown to prevent the aspirin-induced gastric ulcer formation but does not affect gastric juice, acid production or mucosal prostaglandins (PGs) content in rats^6^. [6]-gingerol (6G) is one of the major active components of fresh ginger^7^, and has been reported to exert antioxidant, anti-inflammatory, and anti-cancer activities^1^. Recent reviews demonstrate the role of 6G in prevention and treatment of gastrointestinal cancer^8^, suggesting 6G as a cancer chemopreventive agent^9^. Besides antiproliferative properties, 6G has also been reported to reduce the formation of HCl/ethanol- or aspirin-induced gastric lesion in rats^6^,^10^. With reported anti-cancer and gastroprotective properties, 6G can serve as a lead compound for the discovery of new anticancer drug.

Aspirin (ASA) is used worldwide to reduce pain and inflammation. Low dose aspirin, commonly defined as 75–325 mg daily, is now used for prevention of cardiovascular events^11^,^12^. Recently, large human studies suggest that low dose aspirin has the ability to prevent colorectal cancers^13^,^14^,^15^. The unique pharmacological properties of ASA derive from its ability to acetylate and irreversibly inactivate cyclooxygenase (COX) enzymes, COX-1 and COX-2^16^. ASA is the only nonsteroidal anti-inflammatory drug (NSAID) that covalently modifies both COX-1 and COX-2 enzymes. COX enzymes exist throughout the body, and convert arachidonic acid to various prostaglandins (PGs) in different tissues. Inhibition of COX-2 relieves pain and inflammation, and blocks the vasodilatory and antiplatelet effects in the vascular wall^17^. Blocking COX-1 provides an antiplatelet effect^18^, but inhibits the gastroprotective effects of PGE_2_ and PGI_2_, thereby predisposing the drug users to gastrointestinal complications, such as gastric ulcers and bleeding^17^. In clinical trials, selective COX-2 inhibitors have been shown to retain pain-killing efficacy while reducing gastrointestinal complications^19^,^20^. However, large clinical studies have found an increased risk of developing cardiovascular condition with the use of selective COX-2 inhibitors^19^,^20^,^21^,^22^. Therefore, rather than selective COX-2 inhibitors, the advancement of new aspirin-like molecules with gastroprotective effects would be a welcome development.

The potential long-term therapy of aspirin-like molecules without deleterious side effects in clinical trials prompted our current investigations. The development of ginger ingredient 6G and aspirin in one molecule has never been reported before. We herein report the design and synthesis of [6]-gingerol aspirinate (GAS). The molecular basis of pharmacological properties of GAS was further probed as well.

## Results

### Synthesis and structural elucidation of GAS and AMS

**Figure 1:**
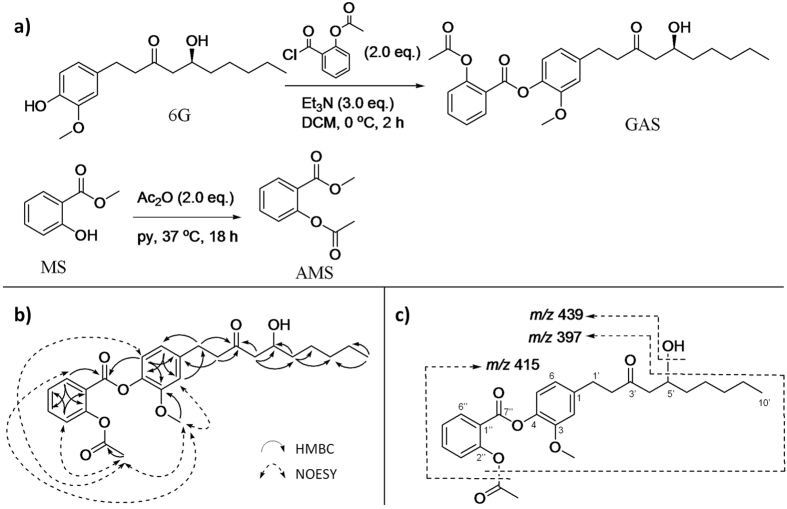
Synthesis of GAS and AMS, and structural identification of GAS. (**a**) Synthetic scheme of GAS and AMS. (**b**) Main HMBC 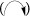 and NOESY 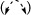 correlations in the structure of GAS. (**c**) Major fragments of GAS in its MS/MS spectra. 6G, [6]-gingerol; GAS, [6]-gingerol aspirinate; MS, methyl salicylate; AMS, acetyl methyl salicylate.

GAS was prepared by acylation of 6G with *O*-acetylsalicyloyl chloride in the presence of Et_3_N at 0 °C (Fig. 1a). The structure of GAS was confirmed by NMR experiments (1D- and 2D-) and mass spectra (Fig. 1b and c). In detail, GAS had a molecular ion at *m/z* 457 [M + H]^+^(295 + 162), indicating a molecular formula of C_26_H_32_O_7_, suggesting that GAS is a [6]-gingerol aspirinate. Aspirin residue and 6G moiety in the structure of GAS were identified by HMBC correlations (Fig. 1b). The attachment of ASA residue at OH-4 in the aromatic ring of 6G moiety through an ester bond was established by a weak HMBC crosspeak between H-5 (*δ*_*H*_ 7.02) and C-7″ (*δ*_*C*_ 162.6, C=O) (Fig. 1b). This linkage was further supported by NOESY correlations observed between OAc-2″ (*δ*_*H*_ 2.29, CH_3_CO) and OMe-3 (*δ*_*H*_ 3.79) as well as H-5 (*δ*_*H*_ 7.02), and between OMe-3 (*δ*_*H*_ 3.79) and H-6″ (*δ*_*H*_ 8.23) (Fig. 1b). Major MS/MS fragments at *m/z* 439 (loss of water), *m/z* 415 (loss of acetyl group), and *m/z* 397 (loss of both acetyl group and water) were also in agreement with the above interpretation (Fig. 1c and Table 1). Thus, the structure of GAS was identified as 4-(5-hydroxy-3-oxodecyl)-2-methoxyphenyl 2-acetoxybenzoate, which is a novel compound.

Acetylation of methyl salicylate (MS) by Ac_2_O in pyridine gave methyl aspirinate (AMS) (Fig. 1a). The structure of AMS was confirmed by interpretation of its ^1^H- and ^13^C-NMR spectra^23^.

### GAS has enhanced anticancer activities in human colon cancer cells than the combination of 6G and ASA, as well as AMS

**Figure 2:**
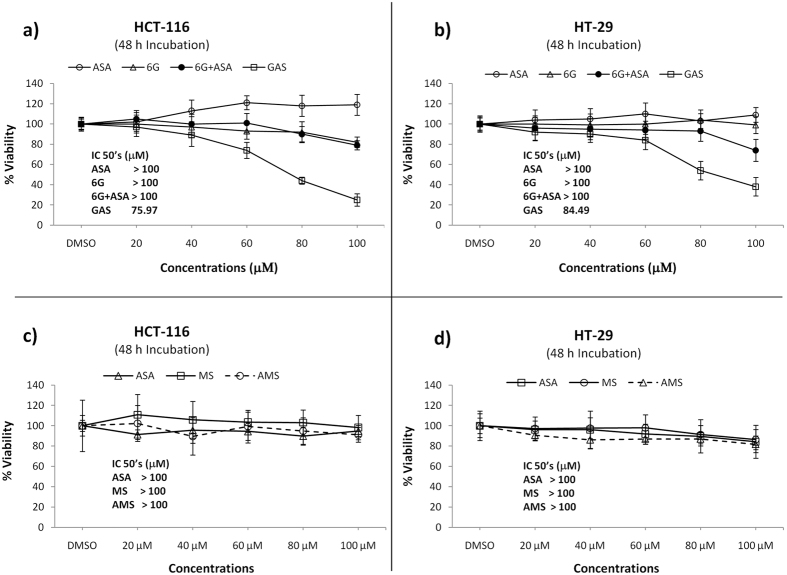
Cell viability by MTT assays. Inhibitory effects of ASA, 6G, 6G + ASA, and GAS (**a**), and ASA, MS, and AMS (**c**) on the growth of human colon cancer cells HCT-116; and of ASA, 6G, 6G + ASA, and GAS (**a**), and ASA, MS, and AMS (**c**) on the growth of human colon cancer cells HT-29. Cells were treated with 20, 40, 60, 80, and 100 μM concentrations of the test compounds for 48 h in the presence of 10% FBS and 1% streptomycin/penicillin at 37 °C. Bar, standard deviation (n = 12). The IC_50_ values are expressed as the mean ± SD (n = 12). ASA, aspirin; 6G, [6]-gingerol; 6G + ASA, an equimolar mixture of 6G and ASA; GAS, [6]-gingerol aspirinate; MS, methyl salicylate; AMS, acetyl methyl salicylate.

**Figure 3:**
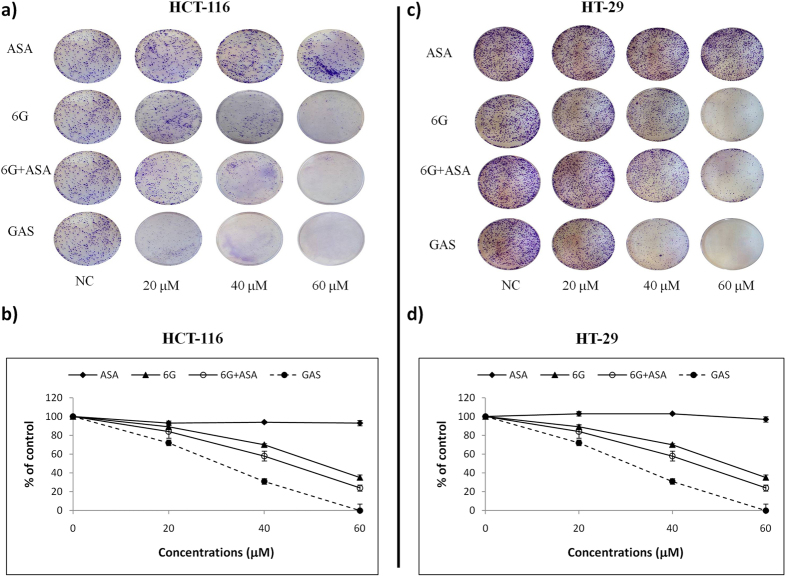
Dose-dependent inhibitory effect of colony formation by ASA, 6G, 6G + ASA, and GAS in HCT-116 (**a**) and HT-29 (**b**) human colon cancer cells. Cells were treated with 20, 40, and 60 *μ*M concentrations of the test compounds and incubated for 2 weeks, and the cells were then stained with crystal violet and counted for colony formation. Each column represents a mean ± SD (n = 3). ASA, aspirin; 6G, [6]-gingerol; 6G + ASA, an equimolar mixture of 6G and ASA; GAS, [6]-gingerol aspirinate.

Subsequently, individual drugs 6G and ASA, an equimolar mixture of 6G and ASA (6G + ASA), and GAS were evaluated for their anticancer activities against human colon cancer cells HCT-116 and HT-29 by MTT assays. As seen, GAS was more active (IC_50_s: 75.97 μM in HCT-116, and 84.49 μM in HT-29) than individual drugs 6G (IC_50_ > 100 μM in both cells) and ASA (IC_50_ > 100 μM in both cells), as well as 6G + ASA (IC_50_ > 100 μM in both cells) (Fig. 2a and b). Likewise, colony formation assays showed that GAS had greater antiproliferative activity than 6G + ASA and individual drugs 6G and ASA, and could inhibit colony formations in both cancer cells in a dose-dependent manner (Fig. 3). In particular, GAS could visually suppress the growth of colonies in cancer cells HCT-116 and HT-29 at concentrations of 20 μM and 40 μM, respectively, compared to control (Fig. 3a and c). At a concentration of 60 μM, GAS nearly completely suppressed the colony formation in HCT-116 by 100% and in HT-29 by 97% (Fig. 3b and d). Whereas, at this concentration, both 6G + ASA and 6G were less active than GAS in both cell lines, and only inhibited the growth of colonies in HCT-116 by 76% and 66%, and in HT-29 by 74% and 68%, respectively (Fig. 3b and d). To decide if gingerol ester in GAS is specifically constitutive of inhibitory effects of this compound on the growth of cancer cells, a simple methyl ester of aspirin (AMS) was synthesized and used along with MS as controls. We found that both MS and AMS exerted similar cytotoxicities to aspirin (IC_50_ > 100 μM in both cells for each), and were less active than GAS (Fig 2c and d). These observations demonstrated that gingerol aspirinate has anticancer potential rather than methyl aspirinate and non-covalent complex 6G + ASA. Our findings identified that GAS has enhanced anticancer properties, suggesting a lower-dose use of GAS in long-term therapy than ASA in clinical trials.

### GAS alleviates gastric ulceration in mice

**Figure 4:**
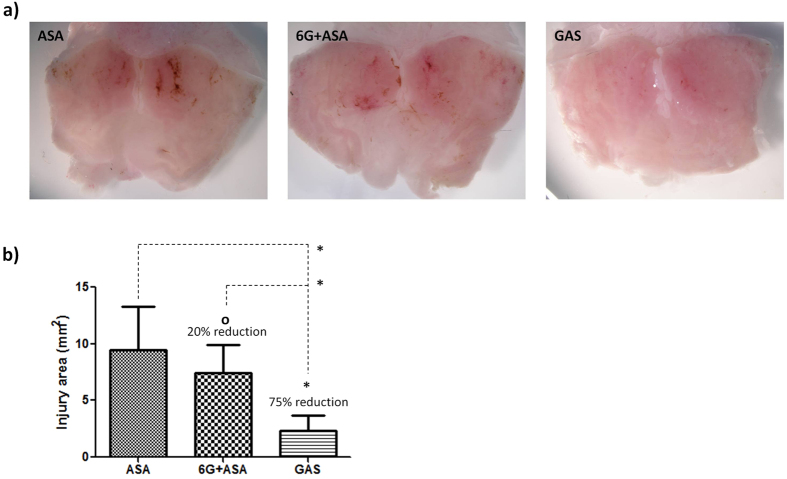
Gastroprotective effect of GAS. (**a**) Photographs of the inner surface of the mouse stomachs treated by ASA, 6G + ASA, and GAS, respectively. (**b**) The gastric mucosal lesion area (mm^2^) measured in each treatment. Results are mean ± SD (n = 3). Bar, standard error; o, not significant; *p < 0.05. All statistical tests are unpaired Student’s *t* tests, two-tailed, compared to positive control (ASA). ASA, aspirin; 6G + ASA, an equimolar mixture of 6G and ASA; GAS, [6]-gingerol aspirinate.

To investigate if GAS effectively reduces the common side effects of ASA, we determined acute gastric lesions and bleeding following intragastric gavage of ASA, ASA + 6G, and GAS in mice. Photographs of the stomach were taken and digitized. As seen in Fig. 4a, the macroscopic images showed that the hemorrhagic gastric lesions coated with coagulated blood in the ASA administered group were more apparent than the 6G + ASA or GAS treated group, verifying that both GAS treatment and the co-administration of 6G with ASA effectively attenuated lesion formation caused by ASA. The measured areas of gastric lesions further demonstrated that GAS significantly inhibited the ASA-induced stomach injury in mice by 75% (*p* < 0.05), and has better protective effects on stomach mucosa than the combination of 6G and ASA (6G + ASA) (20% inhibition) (Fig. 4b). Our results demonstrate the remarkable gastroprotective properties of GAS. The greater potency of GAS also suggests different pharmacokinetics and biodistribution parameters when administration of 6G and ASA as a single molecule (GAS).

### Metabolism and distribution of GAS in mice

**Figure 5:**
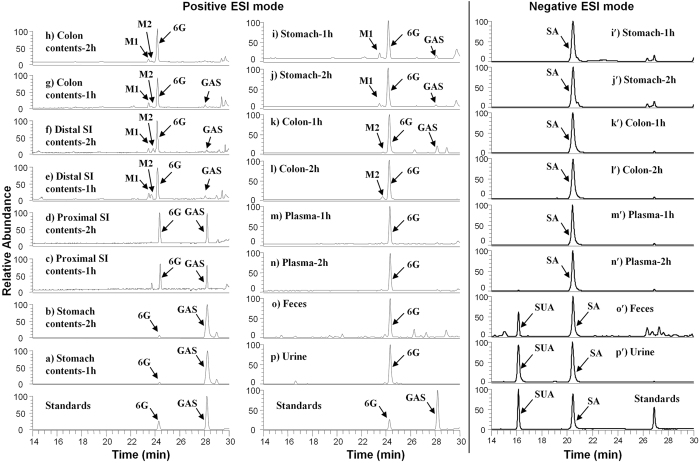
Metabolism of GAS in mice. Extracted ion chromatograms (EIC) of stomach contents collected at 1 h (**a**) and 2 h (**b**), proximal SI contents collected at 1 h (**c**) and 2 h (**d**), distal SI contents collected at 1 h (**e**) and 2 h (**f**), colon contents collected at 1 h (**g**) and 2 h (**h**), colon tissues collected at 1 h (**i**) and 2 h (**j**), stomach tissues collected at 1 h (**k**) and 2 h (**l**), plasma samples collected at 1 h (**m**) and 2 h (**n**), feces (**o**), and urine (**p**) from GAS-treated mice (200 mg/kg, intragastric gavage) obtained by positive ESI-MS interface. EIC of colon tissues collected at 1 h (**i**′) and 2 h (**j**′), stomach tissues collected at 1 h (**k**′) and 2 h (**l**′), plasma samples collected at 1 h (m′) and 2 h (**n**′), feces (**o**′), and urine (**p**′) from GAS-treated mice obtained by negative ESI-MS interface. 6G, [6]-gingerol; GAS, [6]-gingerol aspirinate; M1, (3*R*,5*S*)-[6]-gingerdiol; M2, (3 *S*,5 *S*)-[6]-gingerdiol; SA, salicylic acid; SUA, salicyluric acid; SI, small intestine.

LC-tandem mass spectroscopic data determined the metabolism and biodistribution of GAS in mice. 6G, salicyluric acid (SUA), and salicylic acid (SA) were identified as the major metabolites of GAS in mouse gastrointestinal (GIT) contents, GIT tissues, and biofluids by direct comparison of their respective tandem mass spectra and retention times with authentic standards (Table 1 and Fig. 5). The presence of gingerdiols, (*3R,5S*)-[6]-gingerdiol (M1) and (*3S,5S*)-[6]-gingerdiol (M2), in mice was evidenced by comparing MS/MS fragmentation patterns with published data in our previous studies (Table 1)^24^,^25^. Under positive ESI mode, GAS was found to be almost intact in the stomach, partly hydrolyzed in the proximal intestine and then fully decomposed in the distal intestine to release 6G and ASA in 2 h after oral administration (Fig. 5a–f). 6G was further transformed into gingerdiols M1 and M2 in the distal intestine and colon (Fig. 5e–h), and the remaining 6G was transited out of body through feces (Fig. 5o). Likewise, 6G was extensively distributed in the tissues of stomach and colon (Fig. 5i–l), and predominantly presented in plasma in 2 h after treatment (Fig. 5m and n). On the other hand, SA, a major metabolite of ASA^25^, was abundant in the tissues of stomach and colon, plasma, feces, and urine under a negative ESI mode (Fig. 5i′-p′). Meantime, we found SUA, another metabolite of ASA^25^, in the feces and urine samples (Fig. 5o′-p′). Our results demonstrate that GAS can survive stomach acid and is decomposed to simultaneously release ASA and 6G in intestinal linings. These findings suggest that GAS has resistance to disintegration in the stomach, thereby causing less upper-gastrointestinal bleeding (UGIB) than plain ASA. Also, the departed 6G may simultaneously attenuate the gastrointestinal complications caused by ASA due to its anti-ulcerative effects^3^,^6^.

### GAS inactivates COX-1 and COX-2

**Figure 6:**
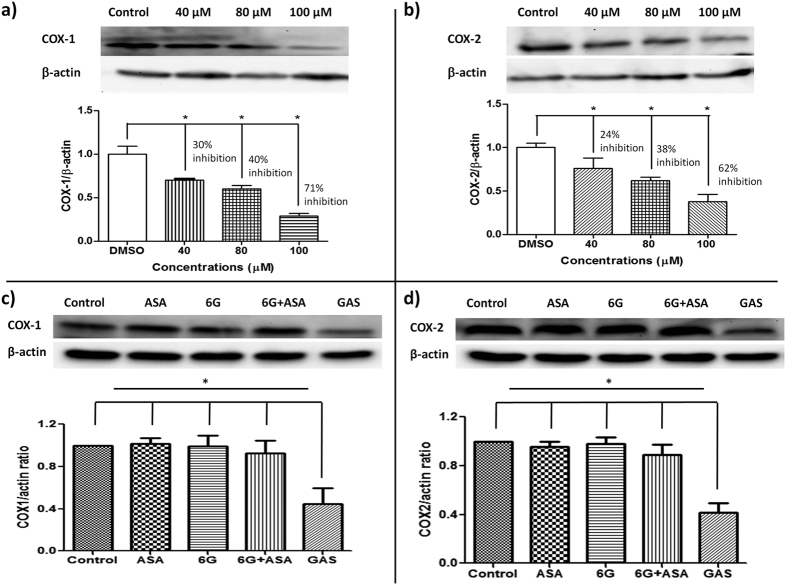
GAS inactivates cyclooxygenases, COX-1 and COX-2. Dose-dependent inhibition of GAS on cyclooxygenases, COX-1 (**a**) and COX-2 (**b**) in human colon cancer cells HT-29. Cell extracts after treatment for 48 h with respective 0, 40, 80, and 100 μM of GAS were performed to Western blot analysis. And inhibitory effects of ASA, 6G, 6G + ASA, and GAS on COX-1 (**c**) and COX-2 (**d**) in human colon cancer cells HT-29, respectively. Cell extracts after treatment for 48 h with respective DMSO, ASA, 6G, 6G + ASA, and GAS at a concentration of 80 μM were harvested for Western blot analysis. Similar results were obtained in three independent experiments. Results are mean ± SD (n = 3). Bar, standard error. *p < 0.05. All statistical tests are unpaired Student’s *t* tests, two-tailed, compared to control (DMSO) or corresponding compounds). ASA, aspirin; 6G + ASA, an equimolar mixture of 6G and ASA; GAS, [6]-gingerol aspirinate.

GAS has been found to enhance anti-cancer properties of ASA while reducing the risk of gastric toxicity in a fashion similar to those selective COX-2 inhibitors^19^,^20^. To determine the selectivity of GAS toward COX enzymes, a Western blotting assay was performed. We found that, unlike selective COX-2 inhibitors, GAS significantly inhibits the expression of both COX-1 and COX-2 in a dose-dependent manner in human colon cancer cells HT-29, compared to the control (DMSO) (*p* < 0.05 for all) (Fig. 6a and b). Particularly, GAS exerted similar inhibitory effects against both COX-1 and COX-2 at tested concentrations (30% vs 24% at 40 μM; 40% vs 38% at 80 μM; and 71% vs 62% at 100 μM) (Fig. 6a and b). Furthermore, Western blotting assays also demonstrated that GAS exerted significantly inhibitory effects towards COX-1 and COX-2 at a concentration of 80 μM, when compared to individuals ASA and 6G, and the mixture of 6G and ASA (6G + ASA) at a same concentration, respectively (*p* < 0.05 for all) (Fig. 6c and d). Our results demonstrate that GAS is a nonselective NSAID. The sustained inhibition of COX-1 in nonselective NSAIDs provides an antiplatelet effect, which may partly reduce the cardiovascular risk associated with COX-2 inhibition, such as Naproxen^18^, implicating the advantages of “balanced” inhibitions of COX-1 and COX-2 in nonselective NSAIDs.

## Discussion

To our knowledge, this is the first study to incorporate 6G, the pungent principal of fresh ginger with gastroprotective effects^6^,^10^, into ASA moiety to form one molecule. We found joint use of non-covalent complex of 6G and ASA (6G + ASA) could significantly suppress the acute injury of stomach induced by ASA in mice, which is in agreement with the important role of 6G in stomachic medications in previous studies^6^,^10^. We further demonstrated that the covalent complex of 6G and ASA (GAS) exerted significantly improved gastroprotective effects than 6G + ASA in mice. The enhanced gastroprotection of GAS could be a result of several contributions. Firstly, GAS survives mouse stomach acids, thereby decreasing the risk of UGIB. Secondly, GAS was decomposed to simultaneously release 6G and ASA in the mouse intestine or after absorption, and concomitant 6G in mouse organs, tissues, and plasma would immediately restore or prevent the local injury caused by ASA. In addition, small amounts of GAS in the tissue of mouse stomach and colon may, at least, exert its own gastroprotection.

Our findings also showed the improved anticancer activities of GAS compared to ASA, 6G, and 6G + ASA, in human colon cancer cells. This observation could be due to the structural skeleton composed of 6G and ASA with higher anticancer properties than those of individuals, or 6G + ASA, similar to our previous findings on the combination of resveratrol and ASA^25^. AMS and MS failed to strengthen the anticancer activity of aspirin, suggesting that methyl ester makes little contribution to aspirin while gingerol ester is a specific building block of GAS for various beneficial physiological effects of aspirin. It is quite understandable that, without gingerol ester in the structure, methyl aspirinate has no ability to reduce the risk of gastric toxicity caused by aspirin, unlike gingerol aspirinate (GAS). In addition, when 6G and ASA were co-administrated (6G + ASA), multiple aspects, including drug interactions, the definitive exposure to the desired targets, and individual pharmacokinetics and biodistribution, should be concerned. These factors may also affect the pharmacological properties of 6G + ASA as a joint use. In contrast, the use of GAS could apparently overcome these difficulties. Our findings suggested that use of an even lower dose of GAS may achieve the equal efficacy of ASA, thereby attenuating the risk of gastrointestinal complications caused by ASA. However, the molecular basis and mechanism of actions of GAS underlying the improved anticancer properties need to be further investigated in the future.

In order to reduce gastrointestinal damage, enteric-coated aspirin and buffered formulations have also been developed to delay the release of ASA beyond the stomach or increase gastrointestinal solubility of ASA in clinical trials^26^,^27^. Results from endoscopic studies carried out in health volunteers indicated that enteric-coated or buffered formulations caused less gastric erosion and microbleeding than plain ASA^28^,^29^,^30^. However, use of low doses of these formulations in patients carries no clear beneficial effects on gastrointestinal symptoms compared to regular aspirin^26^,^27^,^31^. Our findings featured the enhanced anticancer activities of GAS together with significant gastroprotective effects compared to plain ASA. GAS can be an attractive alternative in patients who are at risk of ASA-induced ulceration, as a means of reducing the cardiovascular risk and gastrointestinal complications associated with ASA. Long-term studies estimating clinical GI events are warranted to confirm the clinical GI safety profile of GAS in the future.

## Materials and Methods

### Chemistry

#### General Methods

Reactions were monitored by analytical thin-layer chromatography (TLC) on 250 *μ*m silica gel plates (GF254) (Merck) and visualized under UV light. The products were isolated and purified by column chromatography (CC) using silica gel (Sorbent Technologies). ^1^H and ^13^C NMR spectra were recorded on a Bruker AVANCE 600 MHz spectrometer (Brucker, Inc., Silberstreifen, Rheinstetten, Germany) using TMS as an internal standard. Chemical shifts (*δ*) are expressed in ppm. Coupling constants (*J*) are expressed in Hz, and multiplicities are indicated by s (singlet), d (doublet), t (triplet), q (quartet), and br (broad). The ^13^C NMR spectra are proton decoupled. Aspirin was purchased from MP Biomedicals (Solon, OH). Methyl salicylate was procured from Sigma-Aldrich (St. Louis, MO). 6G was purified from ginger extracts in our lab^32^. Other chemicals were purchased from Sigma-Aldrich (St. Louis, MO) or VWR BDH Chemicals (Radnor, PA), and were used without further purification. All synthesized compounds were>95% pure.

### LC-ESI/MS analysis

LC-ESI/MS was performed with a Thermo-Finnigan Spectra System consisting of an Ultimate 3000 degasser, an Ultimate 3000 RS pump, an Ultimate 3000 RS autosampler, an Ultimate 3000 RS column compartment, and an LTQ Velos Pro ion trap mass spectrometer (Thermo Electrom) equipped with an electrospray ionization (ESI) interface. A Gemini C18 column (5 μm, 3.0 mm i.d. × 150 mm, Phenomenex, Torrance, CA) was employed for the chromatographic separation. The mobile phase consisted of 5% aqueous methanol with 0.1% formic acid (mobile phase A) and 95% aqueous methanol with 0.1% formic acid (mobile phase B). The gradient elution was performed for 45 min at a flow rate of 0.3 mL/min. The gradient was initiated at 0% B and held constant for 3 min, followed by a linear increase to 55% from 5 to 15 min; to 100% from 15 to 30 min, and then held constant for 10 min. The column was then re-equilibrated with 0% B for 5 min. The injection volume of each sample was 10 μL. The LC eluent was introduced into the ESI interface. The positive ion polarity mode was set for the ESI source with the ion spray voltage at approximately 3.5 kV. Nitrogen gas was used as the sheath gas at a flow rate of 34 arb and the aux gas at a flow rate of 10 arb. Optimized parameters, including temperature (300 °C), voltage of the capillary (45 V), and voltage of the tube lens offset (120 V), were tuned using authentic 6G. Selected-ion monitoring (SIM) mode was used to search GAS and its metabolites. Data dependent scanning was performed to obtain the MS/MS spectra. The collision-induced dissociation (CID) was conducted with an isolation width of 1.2 Da and normalized collision energy of 35 for both MS^2^ and MS^3^. The mass range was measured from 50 to 800 *m*/*z*. Data acquisition and analysis were performed using Xcalibur 2.0 (Thermo Electron, San Jose, CA).

### Synthesis of GAS

To a solution of 6G (294 mg, 1.0 mmol, 1.0 eq.) and Et_3_N (0.42 mL, 3.0 mmol, 3.0 eq.) in DCM (5 mL) at 0 °C was added a solution of *O*-acetylsalicyloyl chloride (398 mg, 2.0 mmol, 2.0 eq.) in DCM (2 mL) dropwise. After addition was completed, the mixture was stirred at 0 °C for 2 h, and quenched with water (1 mL) followed by adding 10 mL of ethyl acetate (EA). The organic phase was washed with water (10 mL × 2) and brine (10 mL × 1), dried over Na_2_SO_4_, and filtered. The filtrate was evaporated in vacuo to give a residue, which was purified by CC (H/E = 4:1, 3:1, and 2:1) to get the final product, **GAS**, 450 mg (yield: 99%), as a yellow oil. ^1^H NMR (600 MHz, CDCl_3_) δ 6.82 (1 H, d, *J* = 1.3 Hz, H-2), 7.02 (1 H, d, *J* = 8.0 Hz, H-5), 6.78 (1 H, dd, *J* = 8.0, 1.3 Hz, H-6), 2.90 (2 H, t, *J* = 7.2 Hz, H-1′), 2.78 (2 H, t, *J* = 7.2 Hz, H-2′), 2.59 (1 H, dd, *J* = 17.3, 2.8 Hz, H-4′a), 2.51 (1 H, dd, *J* = 17.3, 9.1 Hz, H-4′b), 4.04 (1 H, m, H-5′), 1.50 (1 H, m, H-6′a), 1.40 (H, m, H-6′b), 1.32 (2 H, m, H-7′), 1.29 (4 H, m, H-8′/9′), 0.89 (3 H, t, *J* = 7.0 Hz, H-10′), 7.16 (1 H, d, *J* = 7.9* *Hz, H-3″), 7.62 (1 H, dt, *J* = 7.9, 1.4* *Hz, H-4″), 7.37 (1 H, t, *J* = 7.9 Hz, H-5″), 8.23 (1 H, dd, *J* = 7.9, 1.4* *Hz, H-6″), 2.29 (3 H, s, CH_3_C=O), and 3.79 (3 H, s, OMe-3); ^13^C NMR (150 MHz, CDCl_3_) δ 140.0 (s, C-1), 112.8 (d, C-2), 151.2 (s, C-3), 137.9 (s, C-4), 122.8 (d, C-5), 120.4 (d, C-6), 29.4 (t, C-1′), 45.0 (t, C-2′), 210.9 (s, C-3′), 49.4 (t, C-4′), 67.7 (d, C-5′), 36.5 (t, C-6′), 25.1 (t, C-7′), 31.7 (t, C-8′), 22.6 (t, C-9′), 14.0 (q, C-10′), 122.6 (s, C-1″), 151.0 (s, C-2″), 124.0 (d, C-3″), 134.4 (d, C-4″), 126.1 (d, C-5″), 132.4 (d, C-6″), 162.6 (s, C-7″), 169.7 (s, CH_3_C=O), 21.0 (q, CH_3_C=O), and 55.9 (q, 3-OMe); positive ESIMS, *m/z* 457 [M + H]^+^.

### Synthesis of AMS

To a solution of methyl salicylate (608 mg, 4.0 mmol, 1.0 eq.) in pyridine (4.0 mL) was added acetic anhydride (816 mg, 8.0 mmol, 2.0 eq.) dropwise. After incubation at 37 °C overnight, pyridine was removed under reduced pressure. The residue was subjected to CC (H/E = 30:1, 20:1, and 10:1) to give AMS (776 mg, yield: 100%), as a white solid. ^1^H NMR (400 MHz, CDCl_3_) δ 7.10 (1 H, dd, *J* = 7.8, 1.1 Hz, H-3), 7.55 (1 H, dt, *J* = 7.8, 1.7 Hz, H-4), 7.30 (1 H, dt, *J* = 7.8, 1.1 Hz, H-5), 8.01 (1 H, dd, *J* = 7.8, 1.7 Hz, H-6), 3.87 (3 H, s, -OCH_3_), and 2.34 (3 H, s, CH_3_C=O); ^13^C NMR (100 MHz, CDCl_3_) δ 123.1 (s, C-1), 150.7 (s, C-2), 123.8 (d, C-3), 133.9 (d, C-4), 126.0 (d, C-5), 131.8 (d, C-6), 164.9 (s, C-7), 52.2 (q, -OCH_3_), 169.7 (s, CH_3_C=O), and 21.0 (q, CH_3_C=O); positive ESIMS, *m/z* 195 [M + H]^+^.

### Biological assays

#### Materials and Methods

Human colon cancer cells HCT-116 and HT-29 were obtained from American Type Tissue Culture (Manassas, VA). McCoy’s 5 A medium was purchased from Thermo Fisher Scientific (Waltham, MA). Supplements of Fetal Bovine Serum (FBS) and penicillin/streptomycin were purchased from Gemini Bio-Products (West Sacramento, CA). Crystal violet, glutaraldehyde and propidium iodide were obtained from Thermo Fisher Scientific (Waltham, MA). Primary antibodies against COX-1 and COX-2 were purchased from Cayman Chemical (Ann Arbor, MI). Secondary antibodies conjugated to HRP (Horseradish Peroxydase) against mouse were purchased from Cell Signaling Technology (Beverly, MA).

### MTT assay

Human colon cancer cells HCT-116 and HT-29 were plated in 96-well microtiter plates with 6000 cells/well and allowed to attach for 24 h at 37 °C. The test compounds (in DMSO) were added to cell culture medium to the desired final concentrations (final DMSO concentrations for control and treatments were 0.1%). After the cells were cultured for 48 h, the medium was aspirated and cells were treated with 200 μL fresh medium containing 2.41 mmol/L 3-(4,5-dimethylthiazol-2-yl)-2,5-diphenyltetrazoliumomide (MTT). After incubation for 3 h at 37 °C, the medium containing MTT was aspirated, 100 μL of DMSO was added to solubilize the formazan precipitate, and plates were shaken gently for 1 h at room temperature. Absorbance values were derived from the plate reading at 550 nm on a Biotek microtiter plate reader (Winooski, VT). The reading reflected the number of viable cells and was expressed as a percentage of viable cells in the control. Both HCT-116 and HT-29 cells were cultured in McCoy’s 5 A medium. The media used above was supplemented with 10% fetal bovine serum, 1% penicillin/streptomycin, and 1% glutamine, and the cells were kept in a 37 °C incubator with 95% humidity and 5% CO_2_. The IC_50_ values were conducted by using GraphPad Prism software (version 5).

### Colony formation assay

Human colon cancer cells HCT-116 (1000 cells per well) were seeded in 6-well culture plates for 24 h, and HT-29 (2000 cells per dish) were seeded in 60 mm cell culture dish for 24 h. The medium were then incubated with the compounds in DMSO in a 37 °C incubator with 5% CO_2_. After 2 weeks, colonies were washed with phosphate-buffered saline (PBS), then stained with a mixture of 6.0% glutaradehyde and 0.5% crystal violet for 30 min at room temperature, rinsed in water, air-dried, and then photographed. The numbers of the colony were counted by using ImageJ software (version 1.48 V).

### Western blot analysis

HT-29 cells were seeded in 145 mm dishes. After 24 h, cells were treated respectively with GAS at 0, 40, 80, and 100 μM for 48 h, and ASA, 6G, 6G + ASA and GAS at 80 μM for 48 h. Total proteins were extracted using ice-cold RIPA lysis buffer [25 mM Tris-HCl (pH 7.6), 150 mM NaCl, 1% NP-40, 1% sodium deoxycholate, 0.1% SDS, Thermo Fisher Scientific] supplemented with a protease inhibitor cocktail (AEBSF, aprotinin, bestatin, E-64, leupeptin and pepstatin A in DMSO with EDTA, Thermo Fisher Scientific). Protein content was measured by a Pierce BCA Assay Kit (Thermo Fisher Scientific). Protein contents of cell lysates (30 μg protein/lane) were resolved by SDS-PAGE. Proteins were then electro-transferred onto PVDF membranes and blots were blocked for one hour at room temperature in 1 × TBS with 1% Casein (Bio-Rad Laboratories, Berkeley, CA). Blots were then incubated overnight at 4 °C with the desired primary antibody, namely, COX-1 (5 μg/ml, 1:200, 70 KDa, Mouse) (Cayman Chemical) and COX-2 (0.5 μg/ml, 1:1000, 72 KDa, Mouse) (Cayman Chemical), diluted in TBS with 0.5% Tween-20. Blots were then washed with TBS-Tween 20 and probed for 1 h with the appropriate secondary antibody HRP (1:1000). Protein bands were visualized with chemiluminescence using West Femto maximum detection substrate (Thermo Fisher Scientific). To confirm equal protein loading in each lane, immunoblots were stripped and re-probed for *β*-actin (1:1000, Rabbit, 45 kDa). Protein fold-induction was calculated by normalizing the intensity of the band of interest to *β*-actin first, and then to control lanes.

### Animal study

Experiments with mice were carried out according to protocols approved by the Institutional Review Board for the Animal Care and Facilities Committee at North Carolina Research Campus (No: 13-014). The methods were carried out *in accordance with* the approved guidelines. Six-week-old female CF-1 mice were purchased from Charles River Laboratories Inc (Durham, NC) and allowed to acclimate for at least 1 week prior to the start of the experiment. The mice were housed 5 per cage and maintained in air-conditioned quarters with a room temperature of 20 ± 2 °C, relative humidity of 50 ± 10%, and an alternating 12-h light/dark cycle. Mice were fed AIN-93G diet from Research Diet (New Brunswick, NJ) and water, and were allowed to eat and drink *ad libitum*.

### Metabolism of GAS

GAS in DMSO was administered to mice by oral gavage (200 mg/kg). Stomach contents and tissue, and intestine contents and tissue were collected at 1 h and 2 h, respectively, after administration of vehicle (control group, n = 5), and GAS (treated group, n = 5). Urine and fecal samples were collected in metabolism cages (5 mice per cage) in 24 h after administration. Blood samples were collected by cardiac puncture and plasma was obtained after centrifugation at 8,000× g for 5 min at 4 °C. The samples were stored at −80 °C until analysis.

#### Sample Preparation

For the metabolic profile study, ~100 mg of each fecal sample (control and treated) was weighted and put into individual tubes. Samples were homogenized with 1.0 mL of MeOH/H_2_O (1:1) with 0.2% AA for 5 min using an Omni Bead Ruptor Homogenizer (Kennesaw, GA) and then centrifuged at 13,200 × g for 10 min at 4 °C. The supernatant (250 μL) was collected and diluted 5 times for analysis. Enzymatic deconjugation of the urine and plasma samples were performed as described previously with slight modifications^33^. In brief, 50 μL of urine or plasma sample from each group (control group and GAS-treated group) were treated with β-glucuronidase (250 U) and sulfatase (3 U) for 24 h at 37 °C. The reaction was stopped by adding an equal volume of MeOH with 1% AA and centrifuged at 13,200 × g for 10 min at 4 °C, then the supernatant was transferred into vials for LC/MS analysis. For the preparation of mouse tissue samples (stomach, small intestine, and colon), 50 mg of thawed tissue was homogenized with 300 μL of sodium acetate buffer (pH 5.4) and then centrifuged at 13,200 × g for 10 min at 4 °C, an accurately measured portion of the supernatant was collected and treated with a mixture of *β*-glucuronidase and sulfatase as described above, the hydrolyzed tissue sample was subjected to LC/MS for analysis.

### Gastric ulceration

The proper amount of ASA, 6G + ASA, or GAS was dissolved in DMSO and ethanol (2% of final volume, respectively) and then suspended in 10% Tween-80 to 2 mL (166.72 mM as final concentration for each tested compound).

#### Animal Treatments

The mice were fasted for 24 h (with as libitum access to water) in metabolic cages and then were administered aspirin (200 mg/kg body weight), 6G + ASA (6G was given to mice in the same molar dose of aspirin), and GAS (GAS was given to mice in the same molar dose of aspirin) suspensions by oral gavage. At 4 h after the administration of test compounds, the mice were sacrificed by euthanization with isofluorane and then blood was collected from the heart. After sacrifice, mouse stomachs were removed and cut open along the greater curvature. The gastric contents were emptied, and the stomachs were then washed with PBS. Gastric mucosal lesions were observed under microscope at 10 × magnification and the size of the all lesions were measured and estimated manually. The inner surface was photographed by Nikon stereoscopic zoom microscope (SMZ1500) with DXM 1200 C digital camera system (0.7 × Relay lens) under Nikon Element Software (Nikon Corporation, Kanagawa, Japan). Next the gastric mucosal tissues were removed, frozen in liquid nitrogen and stored at −80 °C.

### Statistical Analysis

For simple comparisons between two groups, two-tailed Student’s *t-test* was used. A p-value of less than 0.05 was considered statistically significant in all the tests.

## Additional Information

**How to cite this article**: Zhu, Y. *et al*. Gastroprotective [6]-Gingerol Aspirinate as a Novel Chemopreventive Prodrug of Aspirin for Colon Cancer. *Sci. Rep.* **7**, 40119; doi: 10.1038/srep40119 (2017).

**Publisher's note:** Springer Nature remains neutral with regard to jurisdictional claims in published maps and institutional affiliations.

## Figures and Tables

**Table 1:** Major GAS metabolites in GAS-treated mice (200 mg/kg, intragastric gavage) obtained from either positive or negative ESI-MS spectra.

No.	Metabolite^a^	R_t_ (min)	m/z	MS/MS	Occurrence^b^
**1**	GAS	28.25	457 [M + H]^+^	457/439, 415, 397 (B), 379, 319, 257, 177, 163	stomach, small intestine, colon, SC, IC
**2**	6G	24.42	277 [M+H-H_2_O]^+^	277/259, 177 (B), 145	stomach, plasma, urine, feces, SC, IC
**3**	M1	23.12	261 [M+H-2H_2_O]^+^	261/229, 191, 177 (B), 163, 145, 131	urine, feces, colon, IC
**4**	M2	23.54	261 [M+H-2H_2_O]^+^	261/229, 191, 177, 163 (B), 145, 131	urine, feces, colon, IC
**5**	SUA^c^	16.15	194 [M-H]^-^	194/150	urine, feces
**6**	SA^c^	20.48	137 [M-H]^-^	137/93	stomach, small intestine, colon, plasma, urine, feces, IC

^a^GAS, [6]-gingerol aspirinate; 6G, [6]-gingerol. M1, (*3R,5S*)-[6]-gingerdiol; M2, (*3S,5S*)-[6]-gingerdiol; SA, salicylic acid; SUA, salicyluric acid.

^b^CC, colon contents; IC, intestine contents; SC, stomach contents.

^c^Aspirin metabolites, SUA and SA, were identified by negative ESI-MS interface.
